# Viscosity Model for Nanoparticulate Suspensions Based on Surface Interactions

**DOI:** 10.3390/ma14112752

**Published:** 2021-05-23

**Authors:** Benedikt Finke, Clara Sangrós Giménez, Arno Kwade, Carsten Schilde

**Affiliations:** Institute for Particle Technology, Technische Universität Braunschweig, 38106 Braunschweig, Germany; c.sangros@tu-bs.de (C.S.G.); a.kwade@tu-bs.de (A.K.); c.schilde@tu-bs.de (C.S.)

**Keywords:** viscosity, viscosity model, surface interaction, nanoparticle, rheology, complex fluids, nano composite, hybrid modelling, genetic algorithm

## Abstract

In this paper, a widely mechanistic model was developed to depict the rheological behaviour of nanoparticulate suspensions with solids contents up to 20 wt.%, based on the increase in shear stress caused by surface interaction forces among particles. The rheological behaviour is connected to drag forces arising from an altered particle movement with respect to the surrounding fluid. In order to represent this relationship and to model the viscosity, a hybrid modelling approach was followed, in which mechanistic relationships were paired with heuristic expressions. A genetic algorithm was utilized during model development, by enabling the algorithm to choose among several hard-to-assess model options. By the combination of the newly developed model with existing models for the various physical phenomena affecting viscosity, it can be applied to model the viscosity over a broad range of solids contents, shear rates, temperatures and particle sizes. Due to its mechanistic nature, the model even allows an extrapolation beyond the limits of the data points used for calibration, allowing a prediction of the viscosity in this area. Only two parameters are required for this purpose. Experimental data of an epoxy resin filled with boehmite nanoparticles were used for calibration and comparison with modelled values.

## 1. Introduction

Since Einstein’s pioneering work in 1906 [1,2], an abundance of viscosity models have proven the necessity and difficulty of describing the rheological behaviour of suspensions. With the emergence of nano-materials, this field of research has been extended by the need to account for the rheological phenomena caused by nanoparticles immersed in a fluid [3,4].

The production process of nanoparticle suspensions is a typical case in which a viscosity model can be required. During the production of nanoparticle-filled polymers via dispersing of the particles in a resin, the production process itself strongly depends on the viscosity [5]. This is because the viscosity influences the level of stress on the particles during the process. Concurrently, the changes in particle size during the process cause the viscosity to change, which influences the stress level and hence the process. This shows why it is a key prerequisite to be able to model the rheological behaviour of the product suspension, when attempting to model dispersing processes in devices such as kneaders, three-roller mills or stirred-media mills. The viscosity model needs to capture the rheological behaviour based on the shear rate, the particle size and also the temperature, in case it changes along the process. The modelling of dispersing processes most likely requires the viscosity model to be applied numerous times over the course of the modelled dispersing time. It must therefore be designed to require as little numerical effort as possible and focus on the key effects, which cause the rheological behaviour of nanoparticulate suspensions.

A rough distinction can be made among existing models, based on whether they are derived solely on theoretical foundations, rely on some approximated parameters (semi-empirical) or are even based exclusively on a mathematical relations where the parameters do not have a physical meaning (empirical).

Rutgers [6] identified several mathematical approaches, which are used regularly for developing viscosity models. Besides the classical Einsteinian method of accounting for changes in the energy dissipation of a particle-laden fluid under dilation, power law rules, exponential functions and progression formulas have been employed, as they are considered to extend the range of applicability. Another very relevant distinction among the models must be made considering the physical phenomena that they take into account. This is especially relevant for theoretically founded models, since their applicability is usually limited by the narrow boundaries in which assumptions and simplifications can be accepted.

Numerous phenomena are known to influence the rheological behaviour. Jeffrey and Acrivos [7] listed the following: shear rate, diffusion coefficient, surface forces (electrostatic, van der Waals), particle size and particle size distribution, particle shape, particle content, particle Reynolds number (especially critical for coarse particles) and type of flow (linear shear, Couette flow, extensional flow, etc.). Models vary in the number and nature of the phenomena they consider, which reflects the varying significance of the phenomena to the rheological behaviour.

A large number of publications deal with the effect of particles on the rheological behaviour of suspensions. It is apparent that particles alter the rheological behaviour by multiple physical means, with varying effects depending on their dispersion properties and the given flow conditions. Despite their proven relevance, the most commonly used viscosity models, which account for the effect of particles, rely on the solids content as their sole particle-related parameter [3]. The applicability of such simplifications must be considered carefully. A commonly used distinction to limit the applicability of existing theoretical models is whether a suspension is “dilute”, “semi-dilute” or “dense”. Stickel and Powell [8] advocated three conditions that qualify a suspension as being “dense”: The first condition is an interparticle spacing smaller than the particle diameter. The second one is the relevance of both hydrodynamical and surface interaction forces, and the last condition is an overall non-Newtonian rheological behaviour. It must be noted that the latter two conditions follow from the first condition based on the interparticle distance. This is because the hydrodynamic and surface interactions depend on the average interparticle distance and, hence, on the dispersion properties. Therefore, non-Newtonian behaviour can be considered as the phenomenological consequence of it. Thus, a generalized condition to draw a distinction between dilute and semi-dilute suspensions could be proposed by stating that as soon as the interparticle distance is below a limit in which either hydrodynamic forces or surface forces impact the behaviour of the particles, a suspension can no longer be perceived as dilute. Consequently, this limit needs to be identified for every system individually, because the interparticle spacing is a function of the dispersion properties solids content $c_{v}$ and particle size *x*, as will be discussed in detail later. Furthermore, the range, in which surface forces act depends on the type of interaction, as well as the material properties of the particles and the fluid.

It becomes evident that, especially for nanoparticle suspensions, the dilute regime narrows down to very low solids contents and very small windows of material and system parameters. The large number of parameters, which influence the rheological behaviour in the non-dilute regime, has been known for a long time [7] and is reflected in numerous studies dedicated to certain physical phenomena and aspects. However, since the various models only cover one aspect of the physical phenomena at work, none of them provide a universal representation of the rheological behaviour.

Considerable effort has been made to model the rheological properties of colloidal suspensions with respect to the surface forces. Especially the rheological effect of repulsive electrostatic forces has been widely studied. A valuable overview of this field is given in the textbook of Mewis and Wagner [9]. Repulsive forces influence the viscosity, as the diffuse ion double-layer around the particles is deformed when the shear flow forces the particle to move (first electroviscous effect), which increases energy dissipation during flow. When two particles interact via repulsive forces, they evade each other, which again causes stronger energy dissipation during flow and alters the particles’ position (second electroviscous effect). Charged molecules of the fluid can adsorb to the inversely charged surface of the particles and increase the particles’ effective diameter. As these molecules travel along with the particles under shear, they can be considered additional solids content (third electroviscous effect).

Regarding the first electroviscous effect, it allowed Booth [10] to derive an analytical equation for dilute suspensions with low interaction forces. Russel [11] derived an equation based on the second electroviscous effect, which considers the next neighbour distance and the force acting at this point, while neglecting the first electroviscous effect. At the low shear limit, it enables a good prediction of semi-dilute systems [12].

As will be discussed later in more detail, the distribution of neighbouring particles is strongly dependent on the combined effect of surface forces, hydrodynamic forces and Brownian motion. Over the years, the work of Brady, Bossis and Morris has shed much light on the underlying effects. Based on a numerical method called Stokesian dynamics [13], the distribution of neighbours around a particle can be assessed quantitatively [14]. The method even allows depicting the size of particle clusters, which form due to hydrodynamic many-body effects, with respect to flow conditions [15], and how repulsive surface forces change the distribution of neighbours [16]. The method allows the calculation of the viscosity based on the averaging method of Batchelor [17]. Details on the method and the results achieved were discussed in a previous numerical study by the authors [18].

In non-stable suspensions, attractive forces cause particles to flocculate when shear forces are low. The approaching particles interact strongly, which causes extra energy dissipation. Additionally, the fractal structures entrap fluid, which moves along with the particles and artificially increases the volume fraction of the solid phase. Based on a model from Russel [19], several models exist for zero-shear viscosities, which consider the distribution of neighbouring particles and the forces at the respective positions [20,21,22]. Flocculates originating from re-agglomeration of particles can be re-dispersed when sufficient hydrodynamic stress is applied to overcome the particle interactions. This leads to a different spatial distribution of neighbouring particles and hence a different viscosity. Consequently, this is a source of non-Newtonian behaviour. As the build-up and destruction of such flocculates takes time, a time-dependent rheological behaviour (thixotropy) is introduced by instable colloidal particles.

Despite the large body of literature, in many engineering applications, purely empirical models are employed [3]. The majority of these models ignore surface forces or do not capture them in detail. In addition, they consider only the solids content to characterize the disperse phase. This is considered to limit their applicability to dilute systems [3]. In fact, it can be seen that the most successful models [23,24,25,26] consider the particle size as a system parameter. Yet, surface forces are not covered specifically in the models. Among those size-dependent viscosity models for nanoparticulate suspensions, the model of Noni et al. [26] employs the particle size to account for hydrodynamical interactions with respect to the mean distance to the next neighbouring particle ${\bar{a}}_{neigh}$, as given in Equation (1).

(1) $${\bar{a}}_{neigh}=\frac{2}{3}\cdot x\cdot \frac{\left(1-c_{v}\right)}{c_{v}}$$

Based on a force balance applied to a particle, which is immersed in a fluid, Noni et al. yielded the following equation.

(2) $$\eta_{susp}=\eta_{fluid}+k_{1}\cdot {\left(\frac{c_{v}}{1-c_{v}}\right)}^{k_{2}}\cdot \eta_{fluid}$$

Even though surface interaction forces were disregarded explicitly for the derivation of the model [26], when fitting Equation (2) to experimental data, a dependency of the factor $k_{1}$ on parameters influencing the mechanical–geometrical properties could be proven by the authors. This enabled the modelling of the viscosity with respect to the solids content for two material combinations.

In the recent reviews of Mahbubul et al. [27] and Murshed et al. [3] focussing on rheological models employed in the practice of engineering science for nano-suspensions, both concluded that from the very few models specifically designed for the nano-scale, none can describe the rheological behaviour over a broad range of system parameters and no predictive applications are possible. Murshed et al. [3] attributed this finding to the lack of a theoretical foundation of the models.

This paper firstly aimed to outline the fundamental considerations to model the viscosity of a nanoparticle suspension over a broad range of solids contents and shear rates. After that, an incremental method with maximized accuracy is depicted to account for van der Waals forces in a polydisperse system. The effect of surface forces on the viscosity was combined with the influence of further physical phenomena to calculate the viscosity. The required resolution of the incremental method was tested in a convergence study, before applying the method to experimental data and testing its descriptive and predictive abilities. A genetic algorithm was employed to determine some details of the model, as well as to calibrate the model parameters.

## 2. Materials and Methods

### 2.1. Experiments

Experimental data for the comparison with modelled values was gained from a suspension used for the production of fibre-reinforced nanocomposites. Boehmite (AlO(OH)) nanoparticles (Disperal HP 14, SASOL) were dispersed in an epoxy resin (Araldite LY556, Huntsman) via a kneading process, according to a procedure displayed in detail in the work of Jux et al. [28] and subsequently diluted to various solids contents. The epoxy resin showed a Newtonian behaviour and a distinct temperature dependency. The particle size of the suspension was measured via dynamic light scattering (Nanophox, Sympatec, Germany) according to a procedure developed by Nolte et al. [29], which was adjusted to the present material system [28]. Unless stated otherwise in the respective graphs, the particle size (diameter) of the suspension was $x_{50,3}$ = 75 nm.

The rheological characterization was conducted via rotatory viscosimetry in a plate–plate configuration (Kinexus, Malvern) for samples from 1 to 20 wt.%. The measurements were done at fixed shear rates (10–1000 1/s) and temperatures (20–60 °C) to avoid hysteresis from non-steady conditions. The reproducibility of the results was ensured by repeating sample loading and measurements at the extreme points of the highest and lowest shear rate for samples of the neat resin and the suspensions with a solids content of 20 wt.%. Deviations were found to range within 3.5% and 1.8%, which met the required accuracy of the experimental data.

### 2.2. Genetic Algorithm

A genetic algorithm was employed for two important tasks within this study. Appendix A gives a detailed description of the genetic algorithm, its features and the according terminology. The genetic algorithm was first used to aid model development by choosing among weighting factors, whose eligibility could not be determined based on physical considerations. The results are given in Section 3.3. For this purpose, model options with the respective weighting factors were implemented in the code, and the genetic algorithm optimized the probability of execution for each option to come up with the best suited one. For details on these so-called fuzzy genes, see Appendix A. A short presentation of this strategy was given in the review of Thon et al. [30] and set in the context of other methods of artificial intelligence for model development.

Once the model was developed, the genetic algorithm served the purpose of approximating the model parameters. As is outlined in Section 3.3, the settings of the genetic algorithm had to be adjusted to guarantee an efficient performance of the algorithm. Table 1 gives the details on the settings for the respective genetic operators. A large population size of 400 individuals was chosen for the development runs to ensure enough diversity within the population. It was accompanied by strong mutation to further ensure the exploration of the search space.

Once the viscosity model was established, the genetic algorithm was employed for the detailed approximation of the model parameters. A reduced population size of 100 individuals was chosen to reduce the computational effort. No mutation was performed, as heuristic crossover caused enough variation to find the global optimum.

## 3. Model Development

### 3.1. Fundamental Considerations

As mentioned in Section 1, many potential physical phenomena are known to have an effect on the rheological behaviour of a suspension. On this matter, the apparent viscosity is often considered to be the result of the combined effect of these phenomena [7,8,14,31]. Modelling each physical phenomenon and summing their contribution could seem as an approach to model the rheology in the most universal way possible. However, since every modelled aspect comes with simplifications and errors in its parametrization, a large number of approximated parameters would be needed to compensate these effects. Furthermore, the computational effort would be immense, as many models can only be solved analytically for some extreme cases and would require numerical approximation for real-life applications. Limiting the model to the most influential physical phenomena prevents such overflow in approximated parameters.

Consequently, the relevance of the respective phenomena needs to be discussed in order to identify the most relevant ones. Among others, Chang et al. [32] discussed the relevance of several physical phenomena with regard to the parameters solids content $c_{v}$ and shear rate $\dot{\gamma }$. For modelling nano-scale suspensions, the relevance of the individual phenomena needs to be assessed with a focus on particle size, specifically.

All DLVO surface forces depend on both the particle size directly and other properties such as the interparticle distance *b*, which are also affected by the particle size (compare Equation (1)). When discussing London-type van der Waals forces in particular, the rather complex first derivative of the van der Waals potential (see Equation (19)) can be simplified for the monodisperse case to the Derjaguin approximation (Equation (3)), where $r_{i}$ is the particle radius and $W_{vdw}$ is the interaction potential. Further simplification can be made when distinguishing between the two extremes of two interacting spheres in close proximity with $r_{i}>>1/2b$ (Equation (4)) or far-field interactions with $r_{i}<<1/2b$ (Equation (5)), where *b* is the distance between the particles surfaces [33].

(3) $$F_{vdW,i}=\pi \cdot r_{i}\cdot W_{vdw}\left(b\right)$$

(4) $$\begin{array}{ccc} W_{vdW}\left(b\right) & \propto \frac{-1}{b} & for\;r_{i}>>1/2\;b \end{array}$$

(5) $$\begin{array}{ccc} W_{vdW}\left(b\right) & \propto \frac{-1}{b^{6}} & for\;r_{i}<<1/2\;b \end{array}$$

The linear dependence of Equation (3) on the particle radius could be taken to indicate a linear decrease of the van der Waals forces with decreasing particle size. However, at constant solids content, the interparticle distance *b* also depends on the particle size (see Equation (1)). Due to the proportionalities of the van der Waals potential $W_{vdW}$, this effect is compensated (Equation (4)) or even strongly reversed (Equation (5)). Van der Waals forces can therefore be considered very relevant for small particles.

Electrostatic repulsive forces can be described according to the DLVO theory as stated in Equation (6) [34].

(6) $$F_{el}=\kappa \cdot \frac{R}{\nu^{2}}\cdot \frac{32\pi \cdot \epsilon \cdot \epsilon_{0}\cdot {\left(R\cdot T\right)}^{2}}{k_{F}^{2}}\cdot \gamma^{2}e^{-\kappa \cdot b}$$

*R* denotes the gas constant; $k_{F}$ is the Faraday constant; and ν represents the valence of the ions. The parameters γ and κ, *z* and *I* can be described as follows, with $\psi_{0}$ representing the surface potential of the particles and $c_{i}$ the ion concentration:

(7) $$\gamma =\frac{e^{z/2}-1}{e^{z/2}+1}$$

(8) $$z=\frac{\nu \cdot k_{F}\cdot \psi_{0}}{R\cdot T}$$

(9) $$\kappa =\frac{2\cdot k_{F}^{2}}{\epsilon \cdot \epsilon_{0}\cdot R\cdot T}\cdot I$$

(10) $$I=1/2\sum_{i}\left(\nu_{i}^{2}\cdot c_{i}\right)$$

The interparticle distance *b* acts as a negative exponent in Equation (6) and, hence, gives rapid rise to $F_{el}$ as *b* is reduced.

All the above considerations concerning surface forces compel us to regard surface forces when modelling the viscosity of nano-suspensions. They even suggest a dominating role of these effects, especially at low shear rates, where hydrodynamic contributions are comparably small. Surface forces influence the rheological properties of a suspension in several ways. Following Batchelor’s approach [17] by averaging the stress state within a suspension, surface forces act as additional stresslets and increase the stress state. In a recent numerical study, attractive surface interaction forces were proven to induce additional drag forces on nanoparticles [18], which also act as additional stresslets. This phenomenon has long since been postulated for repulsive interactions and was treated analytically by Batchelor [35] for the extreme case of infinite surface forces. Since surface forces impose either attractive or repulsive forces on their neighbours, they have an effect on their neighbours’ relative position, which is counteracted by hydrodynamic forces and Brownian motion. Their relevance to modelling the rheological behaviour of colloidal suspensions was already addressed in Section 1 and must be regarded when modelling the rheological behaviour of nano-scale systems.

Nanoparticles are known to follow the fluid flow closely, as inertia scales to the power of three with the particle size and therefore has only a diminished effect when it comes to the flow state around a particle. The flow state around a particle can be characterized by the Reynolds number (Equation (11)).

(11) $$Re_{particle}=\frac{\rho_{fluid}\cdot v_{rel}\cdot x}{\eta_{fluid}}$$

When taking 100 nm to be the upper boundary for particles to be considered nano-scale and assuming a fluid density $\rho_{fluid}$ in the scale of $10^{3}$ kg/m^3^, $Re_{particle}$ can only reach a value of $Re_{particle}=1$, if either the relative velocity $v_{rel}$ exhibits unrealistically high values (>0.1 m/s) or the fluid viscosity $\eta_{fluid}$ ranges below that of gases (≈10${}^{-5}$ Pa s). The flow state around a nanoparticle immersed in a liquid can therefore be considered to lie in the Stokes regime ($Re_{particle}<0.25$).

The effect of hydrodynamic forces is regarded in many existing models for suspensions with micron-scale particles. Just as surface forces, they represent another contribution (stresslet) to the stress state and also influence the radial neighbour function, which expresses the probability of a neighbouring particle’s presence with respect to the radial distance to the particle centre. See Macdonald [36] for an introduction and comparison of various approaches on defining such neighbour functions. Several phenomena are commonly regarded as hydrodynamic interactions among particles in a sheared suspension. They must be distinguished and regarded individually. One effect is the dissipation of energy due to the rotation of the particles. Quantitative models for this phenomenon are available for spherical [37], as well as ellipsoid particles [38]. Another effect is the emergence of lubrication forces during the approach of particles to each other. Numerous studies strive to model the effect of lubrication forces on the viscosity. Batchelor [17] and Batchelor and Green [39] showed how pairwise hydrodynamical particle interactions affect the rheological behaviour of suspensions. It could be proven, that both, surface roughness [40,41] and Brownian motion [16] affect the rheological behaviour due to lubrication forces. While these effects could only be solved for distinct and simplified cases, numerical simulation allows an assessment of the radial neighbour function in a broader scope [8,14,42]. The interactions are complex, and furthermore, hydrodynamically caused phenomena affect the rheological behaviour of suspensions, such as the many-body dependencies of the interaction [15], as well as ordering and disordering phenomena in the flow [43]. For micron-sized particles, structural effects such as the formation and disintegration of particle clusters are known to yield a strong effect on the energy dissipation during shearing [14,44,45,46,47]. Especially since the structure of such clusters depends on the level of shear stress within the suspension, this phenomenon serves as another cause of non-Newtonian behaviour [48]. At this point, surface interactions also influence the rheological behaviour, as mentioned earlier. Repulsive forces prevent particle clusters from forming or reduce their size [14,16,49], and consequently also alter the overall neighbour function of the particles, which changes the hydrodynamical interaction. The effect of surface forces on the rheological properties of colloidal suspensions was reviewed by Russel [12] and is covered in much detail in the book of Mewis and Wagner [9]. Due to the time-dependent nature of such phenomena, thixotropy arises as viscosity depends on the shearing history of the sample.

The distortion of the radial neighbour function by surface forces and hydrodynamic interactions is counteracted by Brownian motion. The relevance of advective forces on the behaviour of particles is characterised by the particle-related Péclet number Pe, which relates hydrodynamic forces to diffusive forces originating from Brownian motion. The idea is that, while the random movement of the particles due to the diffusive forces allows the particles to reach an energetically stable state, hydrodynamic forces disturb such a state and cause extra energy dissipation during shearing [50]. This effect has been modelled extensively for micron-scale particles [51,52,53]. Brady and Morris [16] used an expression for the Péclet number of particles in shear flow as given in Equation (12).

(12) $$Pe_{particle}=\frac{\dot{\gamma }\cdot r^{2}}{2\cdot D}=\frac{3\cdot \pi \cdot \eta_{fl}\cdot r^{3}\cdot \dot{\gamma }}{k_{B}\cdot T}$$

*D* is the diffusion constant, which can be calculated according to the Einstein–Smoluchowski equation (Equation (13)) with respect to the Boltzmann constant $k_{B}$, temperature *T* and the particle radius *r*.

(13) $$D=\frac{k_{B}\cdot T}{6\cdot \pi \cdot \eta_{fl}\cdot r}$$

Since the particle radius is raised to the power of three in Equation (12), $Pe_{particle}$ rapidly decreases as the particle size is reduced. Therefore, hydrodynamic forces can be considered to have little effect at the nano-scale. Nevertheless, in a numerical study [18] on the viscosity of the same material, hydrodynamic forces were identified to be relevant at high shear rates and high fluid viscosities. This is plausible, as both parameters are in the numerator of Equation (12). While low Péclet numbers indicate a subordinated impact of hydrodynamic forces, surface forces still affect the radial neighbour function, especially at low particle sizes. Maranzano and Wagner [54] asserted the relevance of surface forces to increase linearly compared to hydrodynamic forces based on a dimensionless number they modified from Boersma et al. [55]. Despite a low Péclet number, the diffusion constant in a highly viscous fluid is still low and the influence of Brownian motion limited, as Chen et al. [56] proposed with regards to the temperature-dependent behaviour of nanoparticulate suspensions. Despite the extensive effort to model the impact of various physical effects on the neighbour function, the influence of attractive forces has not been given much focus [12], and repulsive forces have been often simplified to hard sphere interactions. The effect of polydispersity had usually been studied with binary mixtures of particle sizes until Lionberger [49] extended the treatment to Schulze-type particle size distributions.

It becomes clear that for modelling the viscosity of nanoparticulate suspensions, special attention must be paid to interparticle interactions. The flow state around the particles can be regarded as Stokes flow, and hydrodynamic interactions only play a significant role under extreme conditions. A mechanistic representation of hydrodynamic effects and the complex interactions caused by Brownian motion comes at a large expense and varies significantly for every material system. Yet, its relevance cannot be disregarded entirely. In an effort to reduce the modelling complexity, a heuristic expression based on shear rate, particle size and viscosity should be developed, while modelling the effect of surface forces as mechanistically as possible.

### 3.2. Derivation of Model Equations

**Figure 1:**
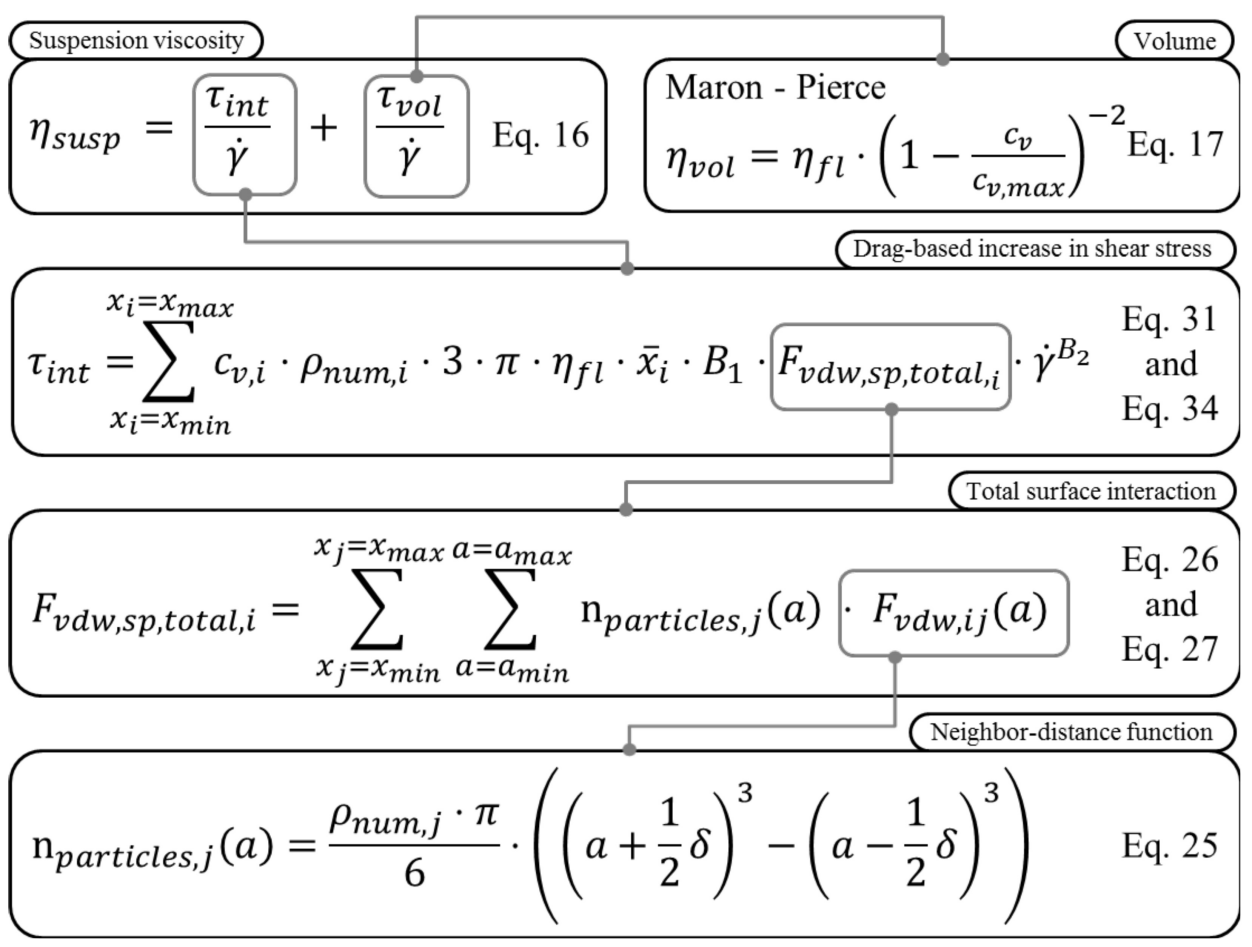
Overview of the viscosity models’ system of equations.

In the fundamental experiments of rheology, fluid is sheared between a steady and a moving plate. The fluid presents a laminar flow regime with a linear velocity gradient. When two small particles are immersed in different lamina of such a fluid, in the equilibrium state, they will follow the stream lines of their respective layer in the laminar flow as long as gravitational forces do not affect their behaviour. Due to interaction forces between their surfaces, the particles experience either attractive or repulsive forces. Coupled CFD-DEM simulations of the same nanoparticulate suspension conducted in a preceding numerical study [18] suggested that in the case of attractive forces, the particle in the slower moving layer of the flow decelerates the faster moving one. In return, the slower particle is accelerated by the particle, which travels faster. As a result, a relative velocity is induced between the particles and their respective surrounding fluid layers. Relative velocities between a body and a fluid cause drag forces. In order to shear a fluid element, these drag forces need to be exerted in addition to the force required to move the pure fluid, which is translated into an increased viscosity. The subsequent sections outline how these relationships are represented and quantified. Figure 1 gives an overview on the system of equations, highlighting their interlinkage.

#### 3.2.1. Model Framework and Adopted Sub-Models

As mentioned above, the apparent viscosity of suspensions is often considered to be the result of additive physical phenomena [7,8,14,31], which increase the overall stress state in the suspension during shearing. Following the same approach, the presented model assumes two additive contributions to the shear stress $\tau_{susp}$ exhibited by the suspension, as presented in Equation (14). This approach already proved successful in the previous numerical study [18]. In Equation (14), $\tau_{int}$ represents the increase in shear stress due to particle interactions, while $\tau_{vol}$ characterizes the increase due to a hindered fluid flow, which is caused by the particles.

(14) $$\tau_{susp}=\tau_{int}+\tau_{vol}$$

According to the fundamental relationship of rheology (Equation (15)), which considers the viscosity η as the proportionality factor between shear stress τ and shear rate $\dot{\gamma }$, Equation (14) can be rewritten as given in Equation (16).

(15) $$\tau =\eta \cdot \dot{\gamma }$$

(16) $$\eta_{susp}=\frac{\tau_{int}}{\dot{\gamma }}+\frac{\tau_{vol}}{\dot{\gamma }}=\eta_{int}+\eta_{vol}$$

$\eta_{vol}$ represents the effect the presence of particles has on the viscosity, simply because they occupy part of the system and alter the fluid flow. This effect is covered by Einstein’s [1,2] equation or its derivatives towards higher solids contents by Mooney [57], Maron and Pierce [58] or Krieger and Dougherty [59].

Within this study, the model of Maron and Pierce (Equation (17)) was chosen to compute $\eta_{vol}$ with respect to the volumetric solids content $c_{v}$, the fluid viscosity $\eta_{fl}$ and the maximum solids content $c_{v,max}$.

(17) $$\eta_{vol}=\eta_{fl}\cdot {\left(1-\frac{c_{v}}{c_{v,max}}\right)}^{-2}$$

$c_{v,max}$ represents the maximum solids content at which the flow of the suspension is possible. The exact value depends on the particle size distribution, and consequently, several values ranging from 0.52 up to 0.74 were found for this parameter [8]. In many cases, $c_{v,max}$ is even used as an adjustable parameter, which deprives $c_{v,max}$ of its physical meaning and even nullifies the models’ physical foundation, when very small values are reached. A value of $c_{v,max}$ = 0.64 was chosen for this study, which corresponds to the maximum random packing of mono-disperse spheres [60].

The fluid viscosity depends on the material and the temperature. In the case of epoxy resins, the curing degree also has an effect. Both empirical and semi-empirical models are available to describe this dependency. The most common ones along with the methods for their parametrisation were recently summarized and contrasted by Abliz et al. [61]. Modelling of the neat fluid viscosity was considered to be outside the scope of this study. Hence, the fluid viscosity was taken as an input parameter in the presented model.

The other term, $\eta_{int}$, in Equation (16) represents the effect of particle interactions on the viscosity. As outlined in detail in the next section, it covers both surface interactions, as well as hydrodynamic interactions, as they were found to be closely related in nano-suspensions, as Section 3.1 suggests.

#### 3.2.2. Determination of Total Particle Interaction

The description of $\tau_{int}$ acting in a system based on particle–particle surface interactions was the key element in this study. When considering only van der Waals forces, according to Israelachvili [33], the potential between two arbitrary-sized particles can be described by the Hamaker approach as given in Equation (18). The first derivative of Equation (18) with respect to the distance results in the force acting between two arbitrary-sized spheres as depicted in Equation (19). $r_{i}$ and $r_{j}$ refer to the particle radii. *b* is the distance between the surfaces of the particles, and *A* represents the Hamaker constant.

(18) $$W_{vdW}=-\frac{A}{6}\left[\frac{2r_{i}r_{j}}{(2r_{i}+2r_{j}+b)b}+\frac{2r_{i}r_{j}}{\left(2r_{i}+b\right)\left(2r_{j}+b\right)}+ln\frac{(2r_{i}+2r_{j}+b)b}{\left(2r_{i}+b\right)\left(2r_{j}+b\right)}\right]$$

(19) $$F_{vdW}=\frac{32}{3}\cdot \frac{Ar_{i}^{3}r_{j}^{3}\left(r_{i}+r_{j}+b\right)}{b^{2}{\left(2r_{i}+2r_{j}+b\right)}^{2}{\left(b^{2}+2br_{i}+2br_{j}+4r_{i}r_{j}\right)}^{2}}$$

The Hamaker constant *A* for particles of the same material (1) immersed in a fluid (3) can be calculated as [33]:

(20) $$A=\frac{3}{4}k_{B}\cdot T{\left(\frac{\epsilon_{1}-\epsilon_{3}}{\epsilon_{1}+\epsilon_{3}}\right)}^{2}+\frac{3h\nu_{e}}{16\sqrt{2}}\frac{{\left(n_{ref,1}^{2}-n_{ref,3}^{2}\right)}^{2}}{{\left(n_{ref,1}^{2}+n_{ref,3}^{2}\right)}^{3/2}}$$

$k_{B}$ represents the Boltzmann constant; *T* is the absolute temperature; while ϵ represents the relative permittivity and $n_{ref}$ the refractive index of the respective materials. *h* represents Planck’s constant, and $\nu_{e}$ is the main absorption frequency in the UV spectrum. The first term of Equation (20) covers Keesom and Debye forces from permanent and induced dipolar interactions, respectively. The second term characterizes the force contribution from fluctuating dipoles, named London forces [33].

Besides their dependency on material parameters, van der Waals forces are influenced by the dispersion properties of the system (Equation (19)). The distance between the particles surfaces *b* is influenced by both the solids content $c_{v}$ and the particle size *x*. Beyond that, these parameters also determine the number of neighbours at a specific distance, which influences the total interaction force on the particles.

The following expression can be derived for the particle number density $\rho_{num}$, representing the number of particles per volume, based on the number of particles $n_{particles}$ with a particle volume $V_{particle}$ in the system volume $V_{total}$:

(21) $$c_{v}=\frac{V_{solid}}{V_{total}}=\frac{n_{particles}\cdot V_{particle}}{V_{total}}=\frac{n_{particles}\cdot \frac{\pi }{6}x^{3}}{V_{total}}$$

(22) $$\Leftrightarrow \rho_{num}=\frac{n_{particles}}{V_{total}}=\frac{6\cdot c_{v}}{\pi \cdot x^{3}}$$

Suspensions are rarely monodisperse, especially when they are produced by top-down processes. Polydispersity has been found to influence the viscosity [62], and its effect has been modelled in detail for binary mixtures [49]. To account for arbitrary particle size distributions, all particle sizes need to be regarded in the model. The particle number density for a specific particle size fraction *i* can be expressed as given in Equation (23).

(23) $$\rho_{num,i}=\frac{6\cdot c_{v}\cdot \Delta Q_{3}\left(x_{i}\right)}{\pi \cdot x_{i}^{3}}=\frac{6\cdot c_{v,i}}{\pi \cdot x_{i}^{3}}$$

$\Delta Q_{3}\left(x_{i}\right)$ refers to the contribution of the particle fraction to a volume-based cumulative particle size distribution $Q_{3}\left(x\right)$, as denoted in Equation (24).

(24) $$Q_{3}\left(x\right)=\sum_{i=x_{min}}^{i=x_{i}}\Delta Q_{3}\left(x_{i}\right)=\sum_{i=x_{min}}^{i=x_{i}}\frac{V(x_{i})}{V_{solid}}$$

To account for the force exerted on a particle by its neighbours, an equation needs to be derived that models the number of neighbours with respect to the radial distance to the particle *i*. For this purpose, the mathematical relationship used to derive $\rho_{num}$ (compare Equation (22)) was applied to a shell-shaped volume element around a particle and included in Equation (25).

(25) $$\begin{array}{cc} n_{particles}\left(a\right) & =\rho_{num}\cdot \left(V_{shell,outer}-V_{shell,inner}\right)\\ & =\frac{\rho_{num}\cdot \pi }{6}\cdot \left({\left(a+\frac{1}{2}\delta \right)}^{3}-{\left(a-\frac{1}{2}\delta \right)}^{3}\right) \end{array}$$

**Figure 2:**
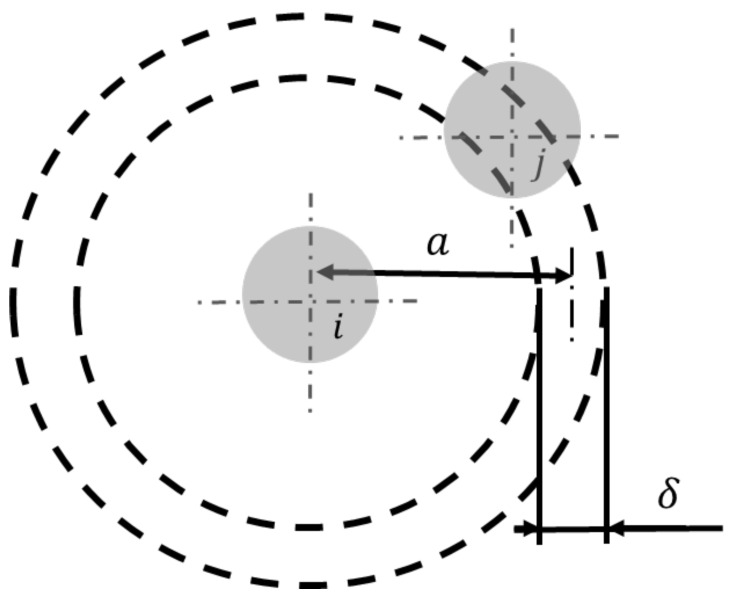
Notation for the derivation of the distance-dependent expression of the particle number $n_{particles}\left(a\right)$.

Equation (25) follows the nomenclature depicted in Figure 2. The distance between the particle centre and the middle of the shell-shaped volume element is denoted as *a*. It is linked to the surface distance *b* via $b=a-(r_{i}+r_{j})$. δ describes the shell thickness. Within the limits of δ, the exact position of the particles centre remains unknown. Therefore, δ can be considered as a resolution parameter to the force evaluation and needs to be chosen sufficiently small to yield converging results.

This procedure represents a simplified approach on specifying the radial neighbour function around particles. Many studies [16,40,51] deal with the task of accounting for influences such as Brownian motion, surface forces, polydispersity and shear rate on this quantity by analytical and numerical means. See for example the work by Lionberger et al. [49] and Stickel [8], as well as the references therein. Disregarding these effects is a simplification, which might limit the applicability of the model and needs to be corrected heuristically. The simplification also prevents capturing time-dependent changes to the neighbour function, which cause thixotropy. Especially attractive surface forces can cause particles to approach and ultimately form clusters. Existing models often capture such behaviour by employing parameters, which specify the formation or degradation of such structures with respect to time, surface forces and the hydrodynamic state. See the textbook of Mewis and Wagner [9] for an overview of this topic. Despite its undisputed relevance to the rheology of colloidal suspensions, such time dependence was not covered in this model, but may be introduced in future work along with a more resolved way of depicting the radial neighbour function.

With the knowledge of the distance-dependent number of neighbours, the force exerted on a single particle (sp) due to the interaction with its neighbour particles $F_{vdw,sp,j}$ can be calculated as depicted in Equation (26). The force evaluation needs to be conducted up to a distance $a_{max}$, whose minimum required value is determined in a later section.

(26) $$F_{vdw,sp,j}=\left|F_{vdw,sp,j}\right|=\sum_{a=a_{min}}^{a=a_{max}}n_{particles,j}\left(a\right)\cdot F_{vdw,ij}\left(a\right)$$

Doing such summation while disregarding the direction of the force implies accounting for the magnitude of forces acting on a single particle, as otherwise, opposing forces would cancel out. The magnitude of forces acting on a single particle was found to be characteristic for the viscosity increase in a previous numerical study [18], which is why it was evaluated here.

In a polydisperse system, the neighbouring particles will vary in size. In order to take this into account, Equation (26) needs to be applied to all particle size fractions in the system, individually. The single contributions add up, as stated in Equation (27).

(27) $$F_{vdw,sp,total}=\sum_{j=x_{min}}^{j=x_{max}}F_{vdw,sp,j}$$

For the present study, only attractive van der Waals forces were considered. The nonpolar epoxy resin prevents an ionic double-layer from forming, which could cause electrostatic repulsion. In principle, such forces could be regarded as well.

#### 3.2.3. Link between Surface Forces and Viscosity Increase

In the numerical study [18], the increase in viscosity due to surface forces was found to result from drag forces acting on the immersed particles. This phenomenon was attributed to the fundamental consideration of two particles immersed in different lamina of a shear flow. The particles are in close proximity, which is why they interact via surface forces. In the case of van der Waals forces, the particles are attracted, and hence, the faster travelling particle accelerates the slower particle, while the slower particle decelerates the other. Thus, they induce a relative motion between themselves and the fluid, which results in the rise of drag forces. These drag forces increase the stress level within the suspension. In the numerical study, the magnitude of the drag force acting on a particle was equal to the magnitude of the total surface interaction force the particle experienced. Accordingly, the shear stress induced by particle interactions $\tau_{int}$ was now considered to be proportional to the drag forces acting on the particles in the system, as stated in Equation (28).

(28) $$\tau_{int}\propto F_{drag,system}$$

In other approaches (e.g., Finke et al. [18]), this relationship is covered in more detail based on stresslets [17]. The present approach did not yield the required information on the direction of the force, but followed the direction-independent proportionality given in Equation (28). Its main benefit was the reduced numerical effort, which must be spent to obtain the stress state.

Due to the very small diameter of nano-sized particles, the particle Reynolds numbers can always be considered to be below 0.25 (compare Section 3.1), which indicates creeping flow, also called Stokes flow. In the Stokes-flow regime, the drag force on a sphere can be expressed as:

(29) $$F_{drag}=3\cdot \pi \cdot \eta_{fluid}\cdot x\cdot v_{rel}$$

Particle size *x* and fluid viscosity $\eta_{fluid}$ were considered to be input parameters to this model. The relative velocity $v_{rel}$ between fluid and particles, however, cannot be accessed easily. Yet, according to the numerical study [18], the magnitude of the drag force and the surface interaction force on each particle must be equal. In order to represent this relationship, the relative velocity was considered proportional to $F_{vdW,sp,total}$ and a proportionality factor $B_{1}$ (compare Equation (30)).

Additionally, the shear rate was found to influence the drag force acting on the particles, according to the numerical study [18]. As this influence was found to be more pronounced at high shear rates and fluid viscosities, it can be considered to be caused by hydrodynamic interactions between the particles. It is well known that also the radial neighbour function of the particles is affected by a complex relationship of shear rate, surface interaction and Brownian motion. Such phenomena were not covered during the determination of total particle interaction in this model. In agreement with the dependency found in the numerical study [18], these effects were represented heuristically by regarding the shear rate based on a power law expression. Hence, the parameter $B_{2}$ was introduced as an exponent to Equation (30).

(30) $$v_{rel}\propto B_{1}\cdot F_{vdW,sp,total}\cdot {\dot{\gamma }}^{B_{2}}$$

Based on these considerations, the impact of the van der Waals force associated with the particle size fractions on the total drag force acting on a single particle $F_{drag,sp,i}$ was regarded by applying the Stokes-flow equation (Equation (29)) and the assumed proportionality (Equation (30)) to the considered particle size fraction *i*. To account for the effect on the shear stress acting on the whole system ($\tau_{int,i}$), $F_{drag,sp,i}$ was multiplied by the number of particles of particle size fraction *i* per volume ($\rho_{num,i}$), as shown in Equation (31). $\bar{x_{i}}$ refers to the mean particle size in of the particle size interval *i*.

(31) $$\tau_{int,i}\propto \rho_{num,i}\cdot F_{drag,sp,i}=\rho_{num,i}\cdot 3\cdot \pi \cdot \eta_{fl}\cdot \bar{x_{i}}\cdot B_{1}\cdot F_{vdw,sp,total,i}\cdot {\dot{\gamma }}^{B_{2}}$$

As $v_{rel}$ is a function of the shear rate $\dot{\gamma }$ to the power of $B_{2}$, the units of $B_{1}$ change, depending on the approximated value of $B_{2}$.

From the contribution of the individual fractions $\tau_{int,i}$, a representative mean value must be calculated. Thus, it must be established how the contributions from the respective fractions must be weighted to obtain the characteristic shear stress $\tau_{int}$ in the system. Plausible options are the weighting according to the number of particles of a particle size fraction with respect to the total number of particles in the system $c_{n,i}$, the solids content $c_{v,i}$ of the size fraction or the contribution of the size fraction to the total particle surface in the system $c_{s,i}$. The three options are displayed in Equations (32)–(34).

(32) $$\begin{array}{cc} \tau_{int} & =\sum_{i=x_{min}}^{i=x_{max}}c_{n,i}\cdot \tau_{int,i} \end{array}$$

(33) $$\begin{array}{cc} \tau_{int} & =\sum_{i=x_{min}}^{i=x_{max}}c_{s,i}\cdot \tau_{int,i} \end{array}$$

(34) $$\begin{array}{cc} \tau_{int} & =\sum_{i=x_{min}}^{i=x_{max}}c_{v,i}\cdot \tau_{int,i} \end{array}$$

The genetic algorithm, which was presented above, was applied to determine the best option, as no option can be ruled out in advance of a test of their applicability. The genetic algorithm was also used to determine the model parameters $B_{1}$ and $B_{2}$ from Equation (30), as well as the parameters from the model for the volume viscosity $\eta_{vol}$ (compare Equation (16)).

Thixotropic effects, which are caused by the shear rate-dependent degradation of flocculates, were not regarded in this model. Still, the question arises of whether the particle size, which is regarded in Equation (31), must be considered shear rate dependent. As this model was developed for epoxy resin suspensions, the high fluid viscosity was considered to fully disintegrate flocculates at very low shear rates. Additionally, the high viscosity strongly reduced the mobility of the particles, slowing down the re-agglomeration during the handling and measurement of the samples to a negligible level. Experimental studies on related suspension systems showed that agglomeration phenomena can only be detected on the time-scale of hours [63], and SEM images of cured samples from the material system addressed in this study did not show flocculated structures either [64]. Consequently, the particle size was considered to be shear rate independent.

### 3.3. Identification of Best Model Variant

A genetic algorithm was employed for model development to choose between three variants of the model, which were hard to assess by other means. As the viscosity model was ultimately meant to depict the monotonic development of shear thinning behaviour, a genetic algorithm may seem rather expensive for a comparably easy optimization problem. The algorithm was mainly chosen for its robustness against converging in local optima. Over the course of model development, the set of equations comprised more parameters, which allowed for an arbitrarily shaped search space, which made the application of a genetic algorithms advisable. Here, the question of which of the functions described by Equations (32) and (33) correctly weights the contributions of each particle fraction demonstrated how the genetic algorithm was used to support the model development, rather than just for a mere approximation of the model parameters.

The genetic algorithm applied the model equations derived in Section 3.2 (see Figure 1 for an overview). At this stage, the objective was to identify the best suited model variant (Equations (32) and (33)), while concurrently optimizing the parameters $B_{1}$ and $B_{2}$. The objective function of the optimization is given in Equation (35). The magnitude of the difference between modelled and the known experimental value was normalized by the experimental value. Normalization was performed to avoid an overly strong fitting to large viscosity values, which can be expected at low shear rates.

(35) $$Error=\frac{|\eta_{model}-\eta_{experimental}|}{\eta_{experimental}}$$

The genetic algorithm was enabled to choose from any of the three options based on the execution probability of the fuzzy gene. Consequently, those individuals were favoured to produce offspring, which exhibited a high execution probability for the particular option, allowing the best representation of the experimental data.

**Figure 3:**
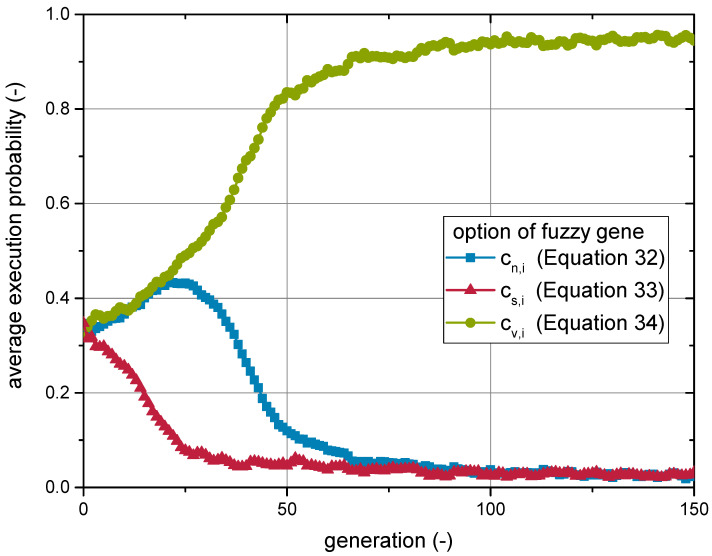
Average execution probabilities of weighting options (Equations (32)–(34)) during the model development run of the genetic algorithm.

In Figure 3, the result of the development run of the genetic algorithm is presented. The execution probabilities are given as an average over the whole population with respect to the number of generations. As outlined above and in Appendix A, the execution probabilities express the likelihood of a model option to be chosen for calculating the viscosity. Large average execution probabilities in the populations implied that a large fraction of the individuals featured a high execution probability for the particular option. Consequently, such an option can be considered to result in a good agreement with the experimental data.

During the first 25 generations, the average execution probability of weighting according to the surface fraction $c_{s,i}$ (Equation (33)) was strongly reduced. Initially, both other options profited nearly equally, while weighting according to the solids content $c_{v,i}$ (Equation (34)) gradually gained dominance over the course of the next 50 generations.

The development of the other parameters ($B_{1}$, $B_{2}$) is not given in detail here. However, it should be noted that, during the subsequent generations, no satisfying agreement between experimental and modelled viscosity values was reached, even after a total of 150 generations. Nevertheless, the general trend, as well as the orders of magnitude of the experimental data were matched, when using the set of parameters (genome) from the fittest individuals. Consequently, an in-depth convergence study for several relevant parameters of the developed model was performed, before calibrating the model in more detail. This is reported in the following section.

## 4. Results and Discussion

### 4.1. Convergence Studies

The viscosity model relied on several parameters, which determined the resolution of the force evaluation with respect to the distance (δ) and the width of the particle size fractions (Δx). In addition, the distance $a_{max}$ up to which the forces were evaluated needed to be specified. Before calibrating the model, convergence studies needed to be carried out in order to ensure that the result was not influenced by the values of these parameters.

**Figure 4:**
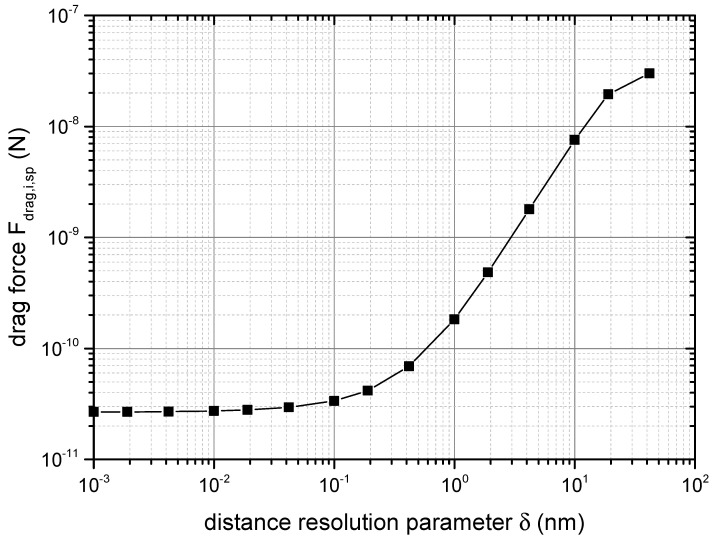
Result of the convergence study on the distance resolution parameter δ according to Equation (25).

Figure 4 displays the effect of the distance resolution parameter δ from Equation (25) on the total acting drag force in the system. It determines the intervals at which the number of neighbouring particles, as well as the force acting between the particles are determined (compare Equations (25) and (26)). By decreasing the magnitude of δ, the estimated force $F_{drag,sp,i}$ was reduced significantly, before converging below a resolution value of 0.02 nm. A reduction of δ increased the runtime significantly. At a value of δ=0.04 nm, the drag force was only changed by 5%, but the runtime was reduced to 50%. In order to profit from this efficiency gain, δ=0.4 nm was used for further work.

**Figure 5:**
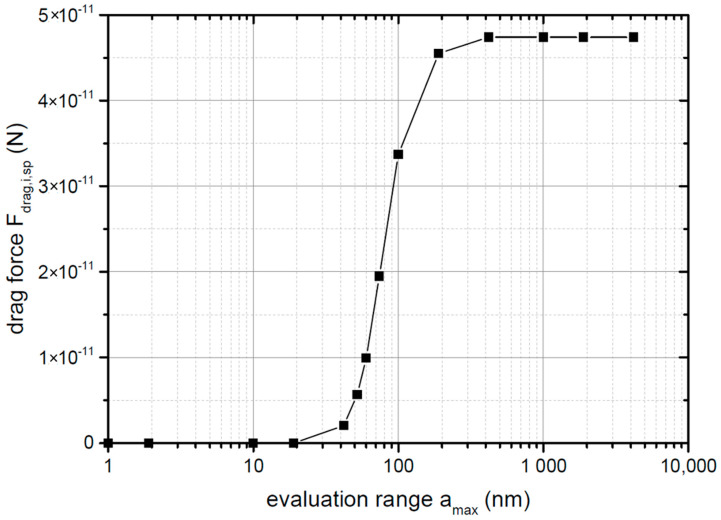
Result of the convergence study on the required range of force estimation according to Equation (26).

The parameter $a_{max}$ specifies the maximum distance to which the force evaluation needs to be performed. Surface forces abated at larger distances while the number of neighbours increased to the power of three (compare Equation (25)). Consequently, at some point, the contributions of the neighbours in the far-field of a particle was so small, that further evaluation did not change the result noticeably. In order to estimate the required distance $a_{max}$, in which the force between the particles needs to be evaluated, $a_{max}$ was gradually increased. The result is displayed in Figure 5, where convergence can be seen above a distance of 400 nm. Again, a reduction of $a_{max}$ to 200 nm only led to a change of 5% in the $F_{drag,i,sp}$ value, which is why this value was chosen to save 50% of the runtime. It must be noted that the determined value for $a_{max}$ cannot be expected to be valid in all cases. The required distance of evaluation may vary significantly, especially when regarding long-range electrostatic repulsive forces. It became evident that despite van der Waals forces usually being assumed to only act over a range of less then 10 nm, the increased number of neighbours extended their effect considerably.

**Figure 6:**
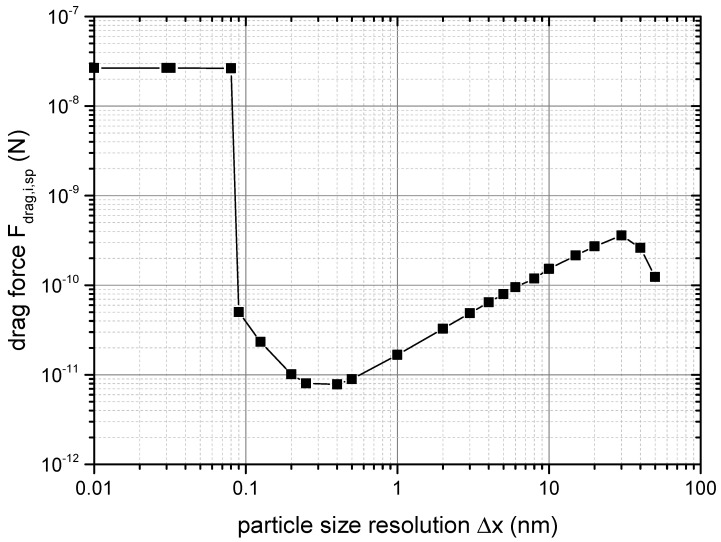
Effect of the particle size resolution Δx on the total drag force acting on a single particle in the system.

To account for polydispersity, the force effect of every particle size fraction in the system was evaluated individually. The question arises of what increment in particle size to choose, in order to achieve representative results. Large increments may result in a poor depiction of the particle size distribution and an insufficiently precise number of neighbours (compare Equation (25)). The effect of the particle size distributions resolution Δx on the drag force acting in the system is depicted in Figure 6. When decreasing Δx, the result changed steadily, until the trend leaped to much higher forces. However, the unchanging region below Δx= 0.08 nm cannot be considered a convergence of the result, as the sharp increase must be attributed to rounding errors. These errors originated from Equation (23), where the values of $\Delta Q(x_{i})$, which correspond to the particle size intervals of Δx, fell below the data types’ precision.

Since no particle size resolution Δx can be justified to be a good choice, Δx= 2 nm was chosen as a trade-off between computational effort and an accurate depiction of the potentially multi-modal particle size distributions. The approximated parameters of the model were sensitive to this value, which was why it needed to be specified along with each set of parameters.

### 4.2. Model Calibration and Assessment of Descriptive Properties

**Figure 7:**
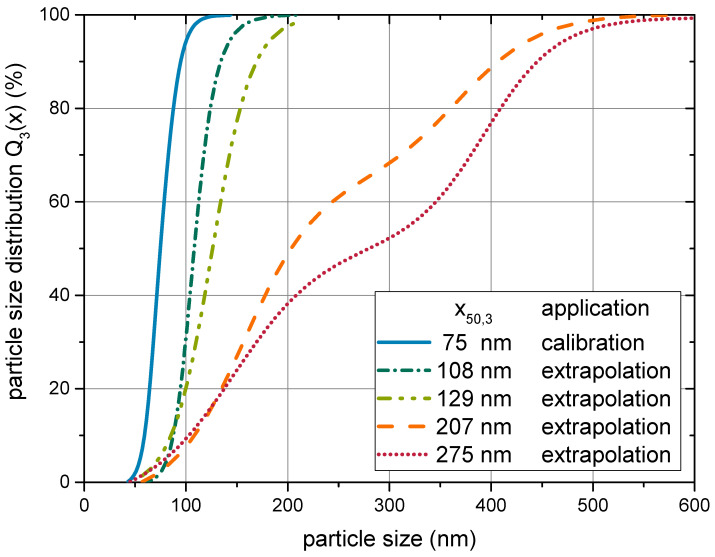
Overview of the particle size distributions of the samples covered in this study. The distribution with $x_{50,3}=75$ nm was used during the model development and calibration of the parameters. The other distributions were applied for evaluating the models’ predictive abilities (see Section 4.3).

The genetic algorithm was applied to approximate the parameters $B_{1}$ and $B_{2}$ for a temperature of 40 °C and a median particle size of $x_{50,3}=75$ nm. The particle size distribution is plotted in Figure 7, along with distribution of samples applied in Section 4.3. Data from shear rate variations between 10 and 10001/s of the suspensions with a solids content from 1 to 20 wt.% were used. The same objective function (Equation (35)) was applied as during the development of the model equations in Section 3.3. Since the best model option was already identified, only the parameters $B_{1}$ and $B_{2}$ were optimized. The calibration yielded the model parameters given in Table 2.

Applying these parameters to any other material system may not be successful, if the system differs strongly from the present one. The first reason is the fact that the parameters correct inaccuracies in the applied material parameters. Especially when the material system requires to regard further surface interactions such as electrostatic repulsion, additional inaccuracies may alter the values of the parameters. Furthermore, the parameters account for deviations of the particles from the assumed spherical shape (compare Equations (19) and (29)), which may also differ in another material system. In addition, the parameters represent the heuristic depiction of the influence of hydrodynamic forces and Brownian motion on the rheological behaviour of the suspension. When other levels of shear rates, particle interactions and shapes are regarded, the physical effects may change, which requires altered parameter values or even a reconsideration of assumptions such as the shear-independent particle size.

**Figure 8:**
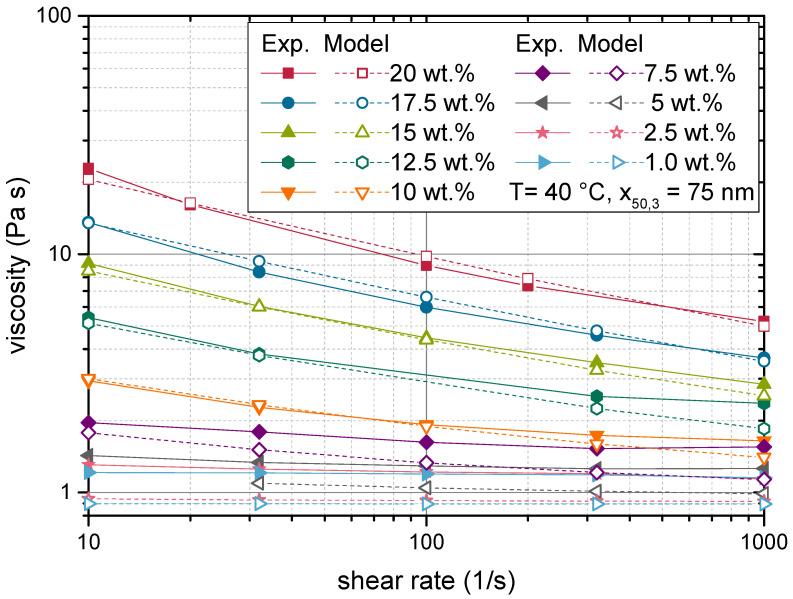
Shear rate dependency of the suspension viscosity at varied solids contents. Comparison of modelled values with experimental data points, which were used for the calibration of the model.

The resulting modelled viscosities are plotted alongside experimental data in Figure 8. A good agreement is observable between experimental and modelled values. Both the increase of viscosity with rising solids content, as well as the shear rate dependent behaviour can be well predicted with the proposed model. The model even succeeded in capturing the change from a Newtonian behaviour at low solids contents to a strong shear thinning behaviour at high solids contents. This indicates that the model covered the essential physical effects that cause the rheological behaviour of nanoparticulate suspensions.

The Péclet number associated with the data points displayed in Figure 8 ranged from 1.2 to 122. This indicates that hydrodynamic interactions had a varying impact on the rheological behaviour of the suspensions and must be regarded in the model. The overall good agreement between modelled and experimental values showed that the heuristic expression of these effects along with the depiction of the shear rate independent contributions regarded in $\eta_{vol}$ was applicable. Furthermore, the effect of Brownian motion on the radial neighbour function can be expected to be less pronounced as might be presumed for nanoparticulate suspensions.

**Figure 9:**
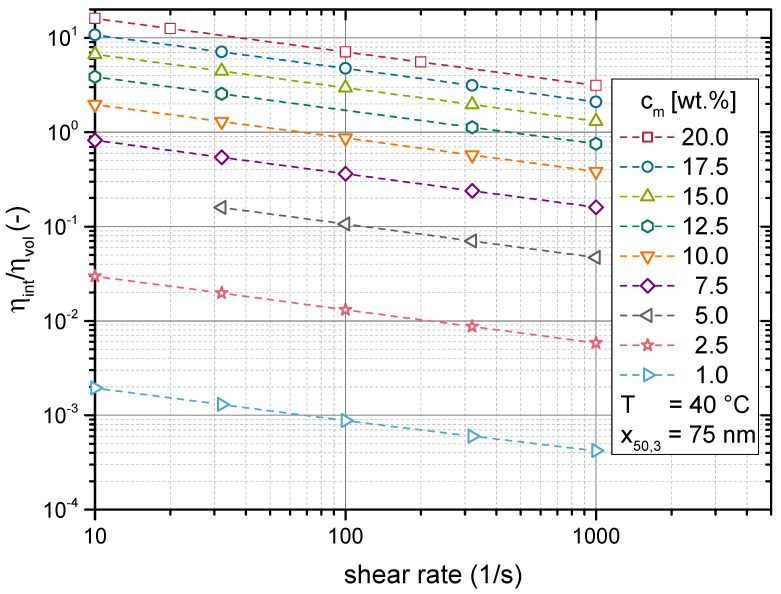
Impact of the respective contributions from Equation (16) on the modelled viscosities displayed in Figure 8. The ratio between viscosity increase due to particle interaction $\eta_{int}$ and the presence of particle volume in the suspension $\eta_{vol}$.

To further evaluate the varying contributions to the model, the impact of the respective viscosity terms of Equation (16) are given in Figure 9 by the ratio of $\eta_{int}$ over $\eta_{vol}$. A ratio of one implies that both terms contribute equally, while higher values indicate a dominating effect of the interaction term $\eta_{int}$. The data presented in Figure 9 suggest that the impact of particle interactions decreased with increasing shear rate. This followed from the fact that the parameter $B_{2}$ was smaller than one (compare Table 2). $B_{2}$ serves as an exponent to the shear rate $\dot{\gamma }$ in Equation (31), which calculates the shear stress due to interactions ($\tau_{int,i}$). This shear stress is then divided by the shear rate (compare Equation (16)) to give the viscosity. Hence, $\eta_{int}$ declines with increasing shear rate. This introduced shear thinning to the suspensions’ rheological behaviour despite the Newtonian behaviour of the neat resin.

It was apparent that especially at low solids contents, the ratio of $\eta_{int}$ over $\eta_{vol}$ was very small. Here, the diminished interaction forces prevented $\eta_{int}$ from having a noticeable effect. Consequently, non-Newtonian behaviour cannot develop at solids contents below 5 wt.%. This region can be considered to represent the dilute regime, as discussed in Section 1.

### 4.3. Predictive Properties

**Figure 10:**
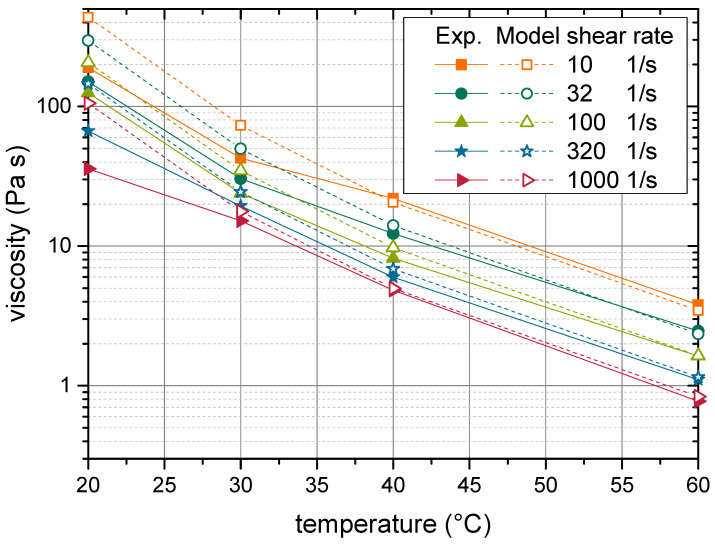
Temperature dependency of the suspension viscosity. Comparison of extrapolated and experimental values. The extrapolation was based on the parameters given in Table 2.

The values of the parameters $B_{1}$ and $B_{2}$, which were obtained from the optimization outlined in Section 4.2 (see Table 2), were used to conduct an extrapolation to other temperatures. With the values for the parameters $B_{1}$ and $B_{2}$ from Table 2, an extrapolation to other temperatures was conducted. The result is depicted in Figure 10. Especially at high temperatures, the model was able to predict the experimental values with good precision. At low temperatures, larger discrepancies arose, and the modelled data reached up to twice the value of the experimental result. Two reasons can be assumed to cause these deviations. At low temperatures, the fluid viscosity is high, and consequently, hydrodynamic interactions gain effect. Hydrodynamic interactions are regarded as a power function in Equation (31). While this expression was already found in numerical simulations [18], the exact mechanism that causes this behaviour is left unknown. Consequently, this effect cannot be modelled with the same mechanistic soundness that was reached for the rheological effect of surface interactions. A far-reaching extrapolation may therefore result in deviations. The second possible cause of deviations may lie in the experimental measurements. Despite the measurements good reproducibility, suspensions with a solids content of 20 wt.% showed poor wettability to the rheometers’ geometry. Hence, wall slip may reduce the measured viscosity.

Regardless of the cause, the observed deviations must be considered with regard to the models’ range of applicability. As is visible from Figure 10, modelled values reached over three orders of magnitude, a range that has not been reached by any present-day viscosity model [3,27]. In view of such an exceptionally broad range of applicability, a mismatch by a factor of two must be considered a very good result.

The fact that there was good agreement between the models’ prediction and the experimental results over a broad range of temperatures indicates that the proposed dependency of the viscosity on the drag force acting on the particles (compare Equation (29)) was correct. Temperature dependency is introduced into the model by the fluid viscosity $\eta_{fluid}$ in Equation (29). Even though Equation (20) indicates a temperature dependency of the Hamaker constant, the temperature-dependent first term of the Hamaker constant only contributed 1.8 % to the total value of the Hamaker constant of the material system. Consequently, a change in temperature hardly affected the Hamaker constant, and the sensitivity of the van der Waals forces to the temperature was minimal. The ability to extrapolate to other temperatures, despite having only used data points at a single temperature for the calibration, shows that the rheological behaviour’s dependency on the drag forces in the system was a sound model foundation.

To this day, a predictive modelling of the temperature dependency of suspension viscosity has not been possible [65]. The same applies for the effect of particle size, especially since it is disregarded in most models in engineering practice [27].

**Figure 11:**
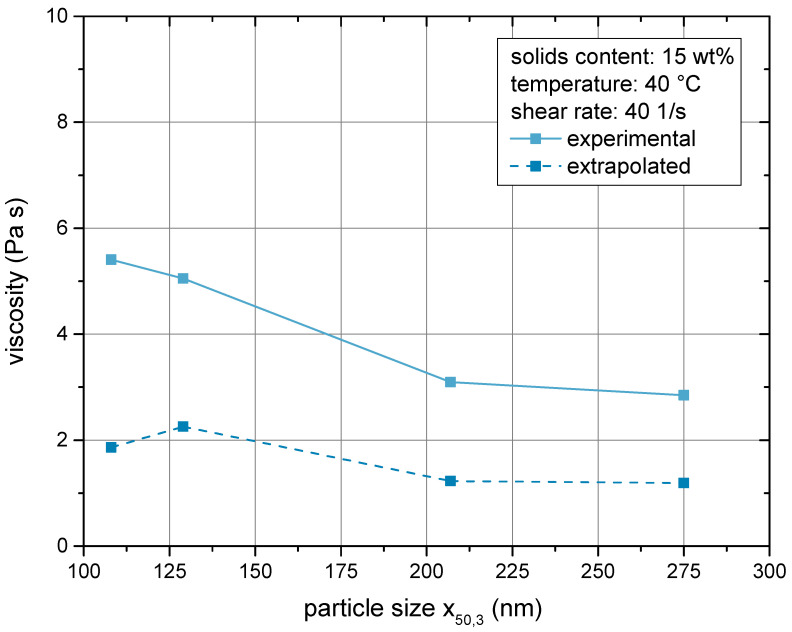
Particle size dependency of the suspension viscosity. Comparison between experimental and extrapolated values. The extrapolation was based on the parameters given in Table 2.

In order to test the ability of the model to depict the particle size dependency of the rheological properties, the model and the calibrated parameters were applied to samples of varied particle sizes. The respective particle size distributions are plotted in Figure 7 along with the distribution used for calibration of the model parameters. It is worth noting that the two coarsest samples exhibited a bimodal particle size distribution, which was regarded in the model. A comparison between the extrapolated values and the experimental results is given in Figure 11. While an offset remained, both graphs followed the same trend. Again, deviations lied in the range of a factor of two. Given the large range of values, in which the model can depict the viscosity, such an offset does not impede the conclusion that the model was capable of capturing the particle size dependency. The offset may also be caused by differences in the particle shape or inner morphology, which cannot be estimated precisely by experiment and was not regarded in the model.

### 4.4. Comparison to Existing Models

A fair comparison of the performance of rheological models is hard to achieve. In many cases, a model’s performance is only determined by whether the assumptions and simplifications made for setting up the model can be held up when looking at a specific material system and experimental conditions. When it comes to modelling the given system, the absence of repulsive forces in the nonpolar epoxy resin prevented most of the existing detailed models from being applicable. The developed equations were based on the assumptions of repulsive surface forces altering the neighbour distribution function.

While analytical solutions can often only be found for the extrema of either a high-shear or low-shear limit, numerical solutions could be applied to cover the range in between. In practical applications, this results in numerous approximation parameters, which deprive the considerations from their physical meaning. The model outlined in this study tackled the problem with a heuristic expression to modify the interaction-based viscosity. In the future, the work of Brady and co-workers could be used to numerically model the interaction among particles, particle forces and fluid and derive simplified characteristic states for a broad range of system conditions. This is especially complicated due to the time dependence induced by the colloidal instability of the system. Yet, using dimensionless numbers, which incorporate surface forces, as well as hydrodynamic forces and Brownian motion, may help with formulating such relationships and even tackle the depiction of time-dependent behaviour.

Krishnamurthy and Wagner [21] developed a model for non-dilute colloidal suspensions with weak attractive forces assuming a square-well potential. In the study, depletion forces caused attractive forces rather than a van der Waals potential. The equation for the relative viscosity $\eta_{rel,0}$ at the lower shear limit is given in Equation (36).

(36) $$\eta_{rel,0}=\frac{\eta_{susp,0}}{\eta_{fl}}=\eta_{rel,0}^{hs}\cdot \left(1+\frac{1.9\cdot c_{v}^{2}}{p_{Baxter}}\right)$$

$p_{Baxter}$ refers to the Baxter parameter or “sticky parameter” to the Baxter potential [66]. When substituting the relative hard sphere viscosity $\eta_{rel,0}^{hs}$ for the model of Maron and Pierce (Equation (17)), the suspension viscosity $\eta_{susp,0}$ at the low shear limit can be calculated as given in Equation (37).

(37) $$\eta_{susp,0}=\eta_{fl}\cdot {\left(1-\frac{c_{v}}{c_{v,max}}\right)}^{-2}\cdot \left(1+\frac{1.9\cdot c_{v}^{2}}{p_{Baxter}}\right)$$

**Figure 12:**
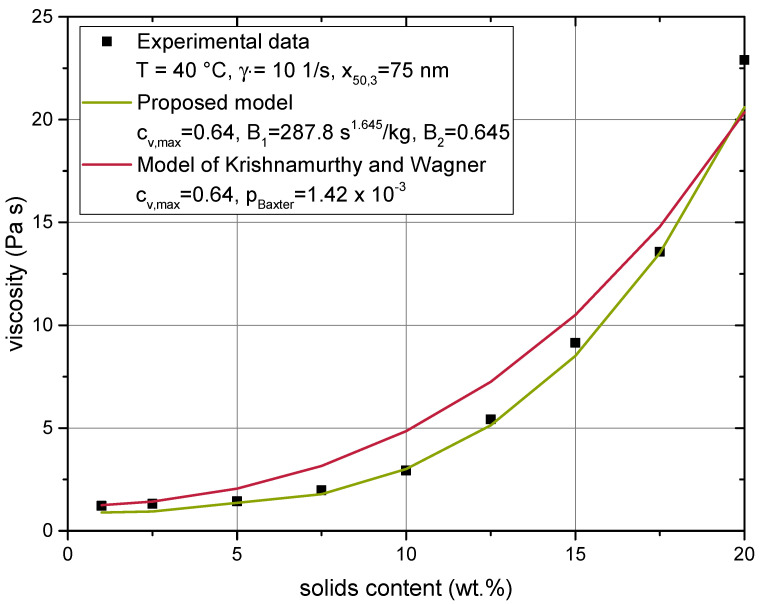
Comparison of the calibrated results of the proposed model (Section 4.2) with the model of Krishnamurthy and Wagner [21].

Figure 12 presents the comparison of the model of Krishnamurthy and Wagner and the proposed model to experimental data. Data from the lowest available shear rate were used for comparison, to meet the assumption of the low shear limit as well as possible. The maximum packing fraction was kept constant at a value of $c_{v,max}=0.64$, as was done during the calibration of the proposed model. Only the Baxter parameter was approximated.

The proposed model depicted the rheological behaviour of the suspension more closely than the model of Krishnamurthy and Wagner. Yet, the performance of the latter model was still very impressive, given that it only featured one adjustable parameter compared to the two parameters of the proposed model. The good performance also indicates that the assumption of weak attractive interaction and its simplification to a square-well potential was not violated much, given the absence of any repulsive forces in the given system. Future work can tell if such simplifications may support computational efficiency to allow for a closer examination of structural and hydrodynamical effects, which influence the neighbour function.

## 5. Conclusions

In this study, a viscosity model was developed, which not only described the rheological behaviour of nanoparticulate suspensions with respect to solids content, shear rate, temperature and particle size, but even allowed an extrapolation far beyond the range of values used for its calibration.

For this purpose, a method to quantify the interaction due to surface forces in a polydisperse system of spherical particles was developed. Based on these interactions, the effect on the suspension viscosity was modelled based on drag forces, which were caused by a change of the particle velocities due to the interaction with other particles.

The high accuracy of the model and its predictive capability suggest that the essential physical phenomena or working principles, which affect the rheological behaviour of the material, were covered. The model was mostly based on system and material properties, which were either known or experimentally accessible. Only two parameters needed to be approximated.

A genetic algorithm was employed to aid the identification of some details of the model equations and the approximation of the parameters. The genetic algorithm was equipped with a novel kind of gene to choose between a variety of model options. A custom method for heuristic crossover was introduced, which promoted the decision-making process.

Future studies should regard all DLVO forces and the effect of particle surface modifications, as this would broaden the range of its applicability. The genetic algorithm could be employed to identify the most suitable models to regard the complex impact of surface forces, hydrodynamic interactions and Brownian motion on the neighbour distribution function of the system. This information could be used to further improve the predictive capabilities of the model. Dimensionless numbers, which express the ratio between particle interactions and hydrodynamic forces, could be a potential tool for a simplified modelling of the complex relationship, as they already proved characteristic for hydrodynamic-driven structural effects [54] and the rheological behaviour of strongly interacting particles [67]. A more resolved way to model the neighbour distribution function based on the above considerations could also be employed to allow for a time-dependent behaviour of the viscosity, which was not covered in the model in its current state.

The presented model opens up the perspective of tailoring nano-scale disperse systems with beneficial rheological properties with significantly reduced experimental effort, as optimum conditions could be extrapolated from few experimental samples.

## Figures and Tables

**Table 1:** Settings used for the genetic algorithm.

Parameter	Development	Calibration
population size	400	100
mutation rate	5%	0%
mutation strength	0.9	−
contrasting parameter *c*	1.2	none
elite fraction	5%	5%

**Table 2:** Optimized parameters of the developed viscosity model. A particle size resolution of Δx=2 nm was used during the evaluation of the particle interactions. The data plotted in Figure 8 were used for calibration.

Parameter		Value
proportionality factor	$B_{1}$	$287.8\;s^{1.645}$/kg
proportionality coefficient	$B_{2}$	0.645

**Figure A1:**
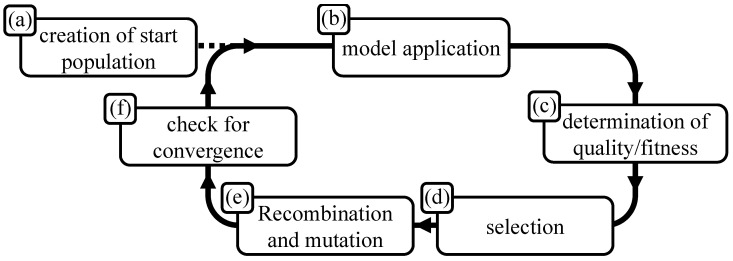
Workflow of the applied genetic algorithm.

## Data Availability

Not applicable.

## Abbreviations

The following Latin symbols and abbreviations are used in this manuscript:

*a*	centre distance
*A*	Hamaker constant
*b*	surface distance
$B_{1}$	approximation parameter to Equation (31)
$B_{1}$	approximation parameter to Equation (31)
$c_{n}$	number fraction
$c_{s}$	surface fraction
$c_{v}$	volumetric solids content
*D*	diffusion constant
*F*	force
*h*	Planck constant
hs	hard sphere
*I*	ion strength
*k*	constant
$k_{B}$	Boltzmann constant
$k_{F}$	Faraday constant
$n_{ref}$	refractive index
$p_{Baxter}$	Baxter parameter [66]
Pe	Péclet number
*r*	radius
rel	relative
*R*	gas constant
Re	Reynolds number
susp	suspension
*T*	temperature
*v*	velocity
*V*	volumetric solids content
*W*	potential
*x*	particle size
*z*	z-parameter

The following Greek symbols and abbreviations are used in this manuscript:

δ	resolution parameter
ϵ	dielectric constant
γ	γ-parameter
$\dot{\gamma }$	shear rate
η	viscosity
κ	Debye–Hückel parameter
ν	valence of ions
$\nu_{e}$	main absorption frequency in the UV spectrum
ρ	density
τ	shear stress

## Appendix A. Principles and Features of the Genetic Algorithm

Genetic algorithms mimic concepts of biological evolution in order to optimize one or multiple parameters. They have proven extremely useful for problems in which the topology of the search space is unknown at the beginning of optimization. If applied correctly, genetic algorithms are very robust against converging to local optima [68,69]. In cases such as the present study, where the model is under development, the search space is unknown and is altered with every change to the set of model equations, which makes the application of genetic algorithms advisable.

In a mathematical sense, a set of parameters is to be optimized during the run of a genetic algorithm. Within the framework of a genetic algorithm, this set of parameters is considered as the genes. The objective is to alter the set of parameters in a way to obtain an optimum result for the given task in analogy to the evolutionary adaption of organisms to environmental conditions. The genes are given a specific order, and the whole set of genes is called a chromosome or an individual in the genetic algorithm. The values of the respective genes are called alleles. They can either be coded as real numbers (floats or integers) or in binary form. Each strategy comes with benefits [70]. In the given study, genes were coded as real numbers for the simplicity of implementation.

The genetic algorithm is comprised of a series of evolutionary operators to achieve the optimization tasks. A very basic and widely used workflow [69,71] was used in this study and is depicted in Figure A1. Initially (a), a start population is created, whose individuals feature random sets of alleles. To evaluate how well the set of parameters performs, the target function, which is the viscosity model in the given case, is then applied with the alleles of the genes (b), and the quality of the result is evaluated (c) by comparison to experimental data. In the subsequent selection process (d), those individuals whose genome gives the best result are favoured. They are chosen to produce offspring, i.e., new parameter sets, by means of recombining the genes/alleles of the parental individuals (e). With a specified probability, mutation occurs during recombination, and the offspring allele is altered. The outlined procedure is repeated, until convergence is reached in the population (f). In the present case, two conditions were evaluated to check for convergence. For each gene, the alleles of all individuals in the population were averaged, and convergence was assumed when this population mean did not change more that 0.01 % over the course of five generations. As a second condition, also the variance in the alleles needed to stagnate within a range of 0.01 % to ensure optimization was completed. When these two conditions were met for every gene, the algorithm ended.

There is a wide variety of ways to design each genetic operator. In the given case, the normalized difference between the model result $\eta_{model}$ and the experimental value $\eta_{experiment}$ was calculated as given in Equation (35). Fitness was calculated by inverting the error according to Equation (A1).

(A1) $$Fitness=\frac{1}{Error}$$

More complex means of weighting errors exist [70], and some were discussed in the work of Jansen et al. [72]. The simple form in Equation (A1) was applied, as complex fitness functions only excel at certain objective functions, and their computational effort can hence only be justified for expert systems after extensive research. Since the given genetic algorithm was applied during model development, the objective function changed multiple times, and such specialisation is not advisable.

Fitness proportional selection was applied to choose individuals for recombination based on how well they performed compared to the rest of the population. It was chosen over other schemes such as tournament selection, truncation selection or ranking selection for its wide usage and proven robustness. Bickle et al. [73] compared these schemes and discussed their respective benefits and characteristics.

Commonly, recombination is done by means of crossover. The chromosome is cut into a specified number of pieces, and the pieces are chosen randomly to form a new chromosome [70]. In the given case, only a small number of parameters need to be optimized, and hence, few genes exist. Few crossover-points would hence result in a random mixing of the genes. The main advantage of this recombination scheme consisting of maintaining a proper parameter combination is therefore negated. Among others, heuristic crossover serves as a good alternative for such scenarios. During heuristic crossover, the offspring’s allele is calculated according to Equation (A2), assuming the allele of parent 1 $w_{parent,1}$ is smaller than $w_{parent,2}$ [70,74].

(A2) $$w_{offspring}=n_{rand}\cdot \left(w_{parent,2}-w_{parent,1}\right)+w_{parent,1}$$

Based on the random number $n_{rand}$, which ranges between zero and one, $w_{offspring}$ can be found between the parental values. An additional benefit of yielding offspring alleles in between the parental alleles is that previously optimized genes more likely remain in a highly desirable region of the search space [70,74,75].

Mutation was implemented in the form of a polynomial mutation according to Deb et al. [76]. A single parameter governs the mutation strength, i.e., the likelihood of mutated alleles to range far from the original value.

Each genetic operator can be influenced by one or more parameters. The choice of parameters is a trade-off between exploitation and exploration, which determines the genetic algorithm’s performance. Exploration describes the algorithm’s ability to cover the search space, while exploitation refers to the algorithm’s efficiency in converging to the right solution. An increased number of individuals in the population along with a large mutation rate and strength increase the explorational power of the algorithm, but delay convergence and, hence, decrease the exploitation. Elitism is a tool to increase exploitation. Here, a fixed number of the fittest individuals passed into the next generation without any alteration, along with the offspring that was very likely generated based on their genome [71].

The genetic algorithm was designed to allow easy modifications of the model such as switching sub-models, adding and removing model parameters and modifying cost functions. This also allows any additional input to be introduced. Such input might serve as an additional constraint or secondary condition to the model.

While genetic algorithms have been employed for a large number of optimization problems [71], they usually evaluate a fixed function. As laid out in Section 2, the genetic algorithm developed in this study was not used just to optimize a fixed set of parameters, but also to develop the viscosity model by choosing between several model options. For this, it allowed numerous mathematical expressions, such as equations or mathematical operators, to be proposed. The genetic algorithm chose among the proposed options, based on a probability of execution assigned to each one. The execution probabilities of all alternatives added up to one and were coded as genes on the chromosome. A randomly generated number decided which option was executed, with highly probable options most likely to be chosen. During selection, those individuals were favoured whose set of model parameters and execution probabilities provided results most similar to the experimental data used for comparison. Hence, individuals that featured a high execution probability for the correct option dominated the population at some point, while inferior solutions went extinct.

The choice of an option might also be done by a Boolean parameter, which dictates whether or not an option shall be executed. When more than two options are presented, an integer parameter could be employed to define which option to choose. However, such approaches come with some disadvantages. Heuristic crossover would not work, as there is no “in between” in Boolean or integer data types. Classical crossover (random choice among chromosomal fragments of the parents for the offspring’s genes) would need to be performed. Furthermore, mutation events would mean a complete reversal of the chosen option. When the rest of the genome is optimized with respect to another option, it is rendered unfit immediately, and the individual is most likely not selected for recombination. A more gradual, in some way fuzzy change among the options allows the rest of the genome to be adjusted.

As described above, the heuristic crossover picks a random value between the parental alleles for each gene. Therefore, the solution is always found between the parental alleles, unless mutation events influence the result. This procedure works well for the approximation of regular parameters, when the search field is chosen sufficiently large. However, fuzzy genes are required to strive for the extremes of either zero or one, which is not found in between the parental alleles. This is why a contrasting parameter *c* was introduced in the heuristic crossover equation for fuzzy genes, as shown in Equation (A3). When *c* is chosen larger than one, high values are increased and low values diminished. This causes results to tend to either of the options over the course of the generations.

(A3) $$w_{offspring}={\left(n_{rand}\cdot \left(w_{parent,2}-w_{parent,1}\right)+w_{parent,1}\right)}^{c}$$

Fuzzy genes require larger populations of individuals, to ensure proper explorational power for every option. The contrasting parameter has a strong effect on the convergence of the fuzzy gene and, hence, on the exploitation ability of the algorithm. At a value of c=2.0, one of the options usually dominates the population after less than 10 generations, with increased risk to converge to a local minimum. Choosing the parameter *c* between one and one-point-five has proven advisable.
